# Organizing pneumonia as the first manifestation of anti-synthetase syndrome

**DOI:** 10.1186/s13104-016-2094-3

**Published:** 2016-06-02

**Authors:** S. M. Thanuja Nilushi Priyangika, W. G. S. G. karunarathna, Isurujith Liyanage, Methsala Gunawardana, Sumeda Udumalgala, Chamith Rosa, Aruna Kulatunga

**Affiliations:** National hospital of Sri Lanka, Colombo, Sri Lanka

**Keywords:** Anti-synthetase syndrome, Interstitial lung disease, Polymyositis, Organizing pneumonia, Anti-JO-1 antibody

## Abstract

**Background:**

Anti-synthetase syndrome associated interstitial lung disease can occur either simultaneously, before, or after the development of polymyositis/dermatomyositis. Histology of interstitial lung disease can be nonspecific interstitial pneumonia, usual interstitial pneumonia, diffuse alveolar damage, organizing pneumonia. Organizing pneumonia associated anti-synthetase syndrome is a rare finding especially as the first manifestation.

**Case presentation:**

We report a 41 year old male patient who presented with organizing pneumonia and 2 years following the onset, developed polymyositis with anti-JO-1 antibody positivity.

**Conclusion:**

It is important to screen patients with organizing pneumonia for anti-synthetase syndrome which can be manifested later.

## Background

Polymyositis (PM) and dermatomyositis (DM) are systemic inflammatory disorders predominantly affecting skeletal muscles and skin respectively. They also affect the oesophagus, lungs and the heart. Pulmonary involvement is common, and may be a major cause of morbidity. It commonly manifests as an interstitial lung disease (ILD) which may progress rapidly and be fatal [1]. Anti-synthetase syndrome is characterized by serum antibodies to aminoacyl-tRNA synthetase and constellation of manifestations, including fever, PM-DM, ILD, arthritis, “mechanics hands”. Organizing pneumonia (previously known as bronchiolitis oblitarence with organizing pneumonia/BOOP) is rarely reported in these patients, as the first presenting symptom [2–4]. We report a case of anti-synthetase syndrome initially presented to us with organizing pneumonia and 2 years later presented as polymyositis.

## Case presentation

**Fig. 1:**
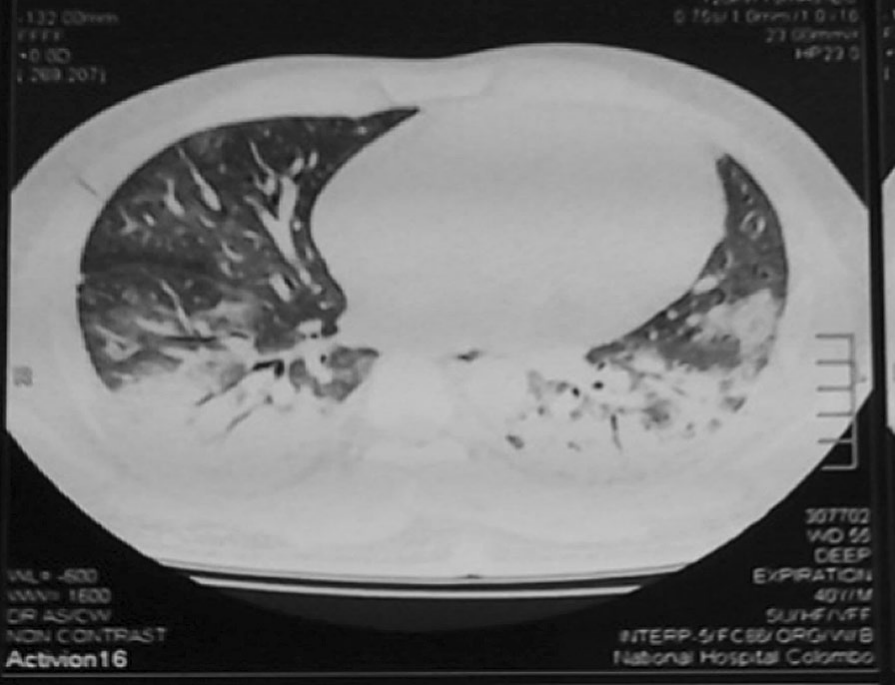
HRCT of the chest showing sub pleural patchy airspace consolidation and ground glass opacification with airbronchogram in bilateral basel regions with sparing of *upper* and *middle* zones, which was compatible with OP

A 41-year-old male with uncomplicated type 2 diabetes mellitus presented with fever and progressive exertional dyspnoea for 1 week. His respiratory system examination revealed bilateral fine end-inspiratory crackles. High resolution computed tomography (HRCT) of the chest showed sub pleural patchy air space consolidation and ground glass opacification with air bronchogram in bilateral basal regions with sparing of upper and middle zones (Fig. 1). Trans-bronchial lung biopsy showed alveoli which are filled with sheets of foamy macrophages with few scattered neutrophils and fibrotic plugs. Alveolar septae appeared thickened. A diagnosis of OP was made based on these findings. During this presentation he did not complain any muscle pain, weakness, joint pains or skin thickening in fingers. He was successfully managed with steroids, which were tailed off after 10 months. Subsequently he was lost to follow up as he failed to show-up at scheduled clinic visits. However 2 years later, he presented with a 2 week history of progressively worsening proximal muscle pain mainly involving the neck and upper limbs. He also had low-grade fever with no skin rashes, arthralgia or respiratory symptoms. There was no history of exposure to dust or medication except metformin. On examination both upper and lower limbs were neurologically normal except proximal muscle tenderness. His respiratory system examination was unremarkable.

**Fig. 2:**
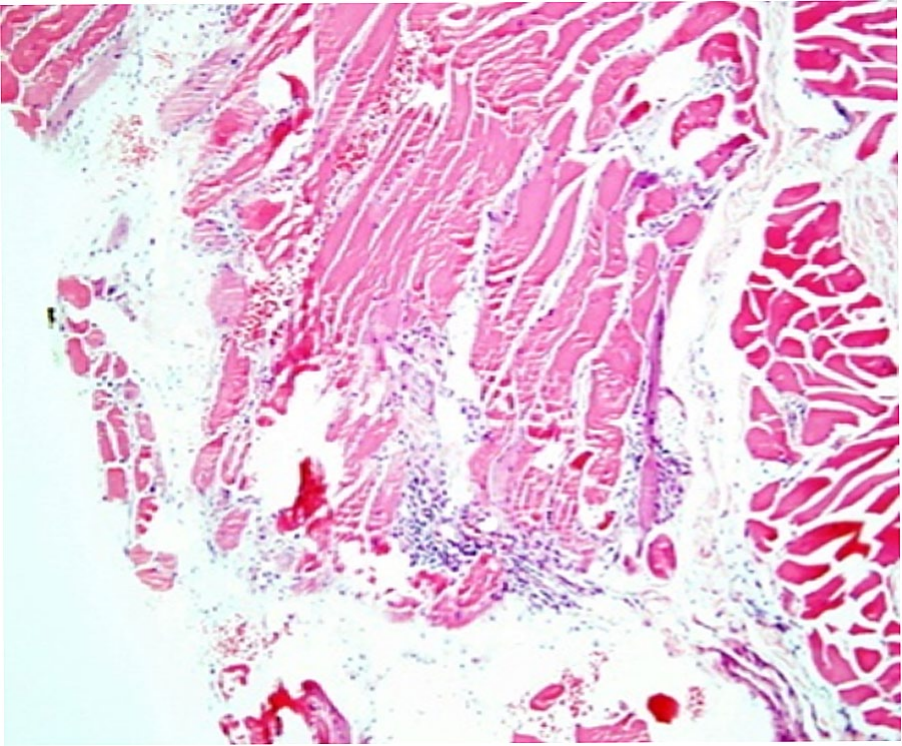
Histology of muscle biopsy showing varying sized muscle fibers with focal degenerative and regenerative changes and a chronic inflammatory infiltrate destroying myocytes (H&E)

Laboratory investigations revealed creatine phosphokinase (CPK) 14820 U/L, C reactive protein (CRP) 110 mg/dL, erythrocyte sedimentation rate (ESR) 70 mm/1st h, thyroid stimulating test (TSH) 1.84 mIU/L and free T4 0.94 ng/dL. The electro-myography (EMG) did not show evidence of myositis or myopathy. Anti-Jo-1 antibody and the anti-nuclear-antibody were positive. Deltoid muscle biopsy (Fig. 2) and magnetic resonance imaging (MRI) were compatible with polymyositis. Repeat HRCT and CXR were compatible with a relapse of an ILD.

Based on the clinical presentation and investigations, diagnosis of anti-synthetase syndrome was made. He was treated with high dose prednisolone and azathioprine. After 1 month of treatment the CPK and inflammatory markers normalized.

## Discussion

Anti-synthetase syndrome is a rare systemic autoimmune syndrome, characterized by the presence of anti-aminoacyl-tRNA antibodies (anti-Jo-1) accompanied by a constellation of clinical findings including PM-DM, ILD, “mechanics hand” appearance, fever and Reynaud’s phenomenon [5–7]. This occurs mainly in adults and more common in females and etiology is not known [6]. The anti-aminoacyl- tRNA antibodies directed toward the attachment of particular amino acid to its transfer RNA (tRNA). There are several anti-synthetase antibodies and anti-Jo-1 is the commonest and occurs in 80 % of patients with anti-synthetase syndrome [8], others are PL-7, PL-12, OJ, EJ [6, 7, 9].

Myositis, ILD and joint involvement are the classic tried in anti-synthetase syndrome. Myositis occurs in more than 90 % of patients and ILD found in more than 60 % of patients. ILD in anti-synthase syndrome is a major cause of morbidity and it can occur in the absence of myositis (amyopathic ILD) [7, 8]. Because of this anti-synthetase antibodies, particularly anti-Jo-1 should be performed in all patients with ILD without an obvious etiology [8]. Identification of anti-synthetase syndrome in patients with amyopathic ILD would be important as there are therapeutic implications [7]. Studies have demonstrated the efficacy of immunosuppressive agents in ILD associated with anti-synthetase syndrome whereas lung transplantation has so far been the only treatment option in idiopathic pulmonary fibrosis [7].

Histology may show different patterns including nonspecific interstitial pneumonia (NSIP), diffuse alveolar damage (DAD), usual interstitial pneumonia (UIP) or organizing pneumonia (OP). The prevalence of these histological features varies between reports and NSIP is the commonest pattern [5, 7].

Although OP is commonly seen with rheumatoid arthritis (RA), manifesting after the onset of arthritis, it is rare with PM-DM and manifests before the onset of myositis as in our patient [3]. However organizing pneumonia complicating polymyositis carries a better prognosis than UIP or DAD [1].

Joint involvement occurs in more than 50 % of patients with anti-synthetase syndrome and it can range from simple arthralgia to arthritis which can be erosive [6]. “Mechanics hands” occurs in 30 % of patients and Raynaud phenomenon occurs in 40 % [7].

Although over 90 % patients with polymyositis typically present with proximal muscle weakness, mild myalgias and muscle tenderness occur in 25–50 % of cases and 11 % of patients show normal EMG as in our patient [10]. Positive anti-Jo-1 antibody, muscle histology and muscle MRI confirmed the diagnosis of polymyositis in this patient.

As our patient had positive anti JO-1 antibody together with fever, PM and ILD the diagnosis of anti-synthetase syndrome was made. At the time of diagnosis Raynaud phenomenon, joint involvement, “mechanics hands” were absent in our patient but these can be manifested later [7].

The presence of Anti-Jo-1 is known to be associated with poor survival, lesser response to steroids and a higher incidence of flare-ups when steroids are tapered off. This patient however responded to steroids and is currently stable on a tail off regime of steroids and azathioprine.

## Conclusion

Organizing pneumonia associated anti-synthetase syndrome is a rare finding especially as the first manifestation. This case signifies the importance of screening patients with OP and other ILD without obvious etiology for anti-synthetase syndrome and arranging long term follow-ups [8].

## Abbreviations

PM: polymyositis
DM: dermatomyositis
ILD: interstitial lung disease
BOOP: bronchiolitis obliterance organizing pneumonia
OP: organizing pneumonia
NSIP: nonspecific interstitial pneumonia
DAD: diffuse alveolar damage
UIP: usual interstitial pneumonia
